# The Effect of Leukocyte- and Platelet-Rich Fibrin on Central and Peripheral Nervous System Neurons—Implications for Biomaterial Applicability

**DOI:** 10.3390/ijms241814314

**Published:** 2023-09-20

**Authors:** Ivo Lambrichts, Esther Wolfs, Annelies Bronckaers, Pascal Gervois, Tim Vangansewinkel

**Affiliations:** 1 Cardio and Organ Systems, Biomedical Research Institute, UHasselt—Hasselt University, 3590 Diepenbeek, Belgium; esther.wolfs@uhasselt.be (E.W.); annelies.bronckaers@uhasselt.be (A.B.); pascal.gervois@uhasselt.be (P.G.); 2 VIB, Center for Brain & Disease Research, Laboratory of Neurobiology, 3000 Leuven, Belgium

**Keywords:** leukocyte- and platelet-rich fibrin, neural stem cells, primary cortical neurons, sensory neurons, neurotoxicity, neuritogenesis

## Abstract

Leukocyte- and Platelet-Rich Fibrin (L-PRF) is a second-generation platelet concentrate that is prepared directly from the patient’s own blood. It is widely used in the field of regenerative medicine, and to better understand its clinical applicability we aimed to further explore the biological properties and effects of L-PRF on cells from the central and peripheral nervous system. To this end, L-PRF was prepared from healthy human donors, and confocal, transmission, and scanning electron microscopy as well as secretome analysis were performed on these clots. In addition, functional assays were completed to determine the effect of L-PRF on neural stem cells (NSCs), primary cortical neurons (pCNs), and peripheral dorsal root ganglion (DRG) neurons. We observed that L-PRF consists of a dense but porous fibrin network, containing leukocytes and aggregates of activated platelets that are distributed throughout the clot. Antibody array and ELISA confirmed that it is a reservoir for a plethora of growth factors. Key molecules that are known to have an effect on neuronal cell functions such as brain-derived neurotrophic factor (BDNF), nerve growth factor (NGF), vascular endothelial growth factor (VEGF), and platelet-derived growth factor (PDGF) were slowly released over time from the clots. Next, we found that the L-PRF secretome had no significant effect on the proliferative and metabolic activity of NSCs, but it did act as a chemoattractant and improved the migration of these CNS-derived stem cells. More importantly, L-PRF growth factors had a detrimental effect on the survival of pCNs, and consequently, also interfered with their neurite outgrowth. In contrast, we found a positive effect on peripheral DRG neurons, and L-PRF growth factors improved their survival and significantly stimulated the outgrowth and branching of their neurites. Taken together, our study demonstrates the positive effects of the L-PRF secretome on peripheral neurons and supports its use in regenerative medicine but care should be taken when using it for CNS applications.

## 1. Introduction

Successful repair of damaged or missing tissue due to trauma, age or disease is the main goal of the field of regenerative medicine and tissue engineering [1]. (Bio)materials constitute an important aspect of current regeneration strategies as they can resemble the natural extracellular matrix (ECM), which provides (transient) structural support and/or directs the endogenous response towards structural and functional repair [2,3]. Moreover, several biomaterials or synthetics that show great promise in tissue repair and regeneration are characterized by the incorporation of growth factors or the combination of growth factors, embedded (stem) cells, and the supportive ECM [3,4,5,6]. In addition to wound healing, several attempts have been made to use a scaffold-based approach for treating peripheral nerve injury (PNI) [7,8,9] and pathologies such as stroke, and traumatic brain or spinal cord injury [10,11,12,13,14]. Unfortunately, several of these treatments remain expensive or experimental and are therefore not suitable for all patients [4,8,13]. Hence, it is of high importance to find new biomaterials that meet these requirements, and that can be used in tissue engineering strategies to restore tissue damage in the peripheral- and central nervous system (PNS, CZS).

Platelet concentrates are innovative tools in regenerative medicine, and different types have been developed over the years, such as traditional platelet-rich plasma (PRP), pure PRP, and leukocyte PRP [15,16,17,18]. Leukocyte- and Platelet Rich Fibrin (L-PRF), on the other hand, is a second-generation platelet concentrate that was first described in 2006 by Choukroun et al. [19,20]. It is characterized by a dense fibrin network (resembling the natural fibrin clot) and it is rich in platelets, leukocytes, growth factors, and many other (inflammatory) mediators (e.g., ATP, serotonin, cytokines) [21,22,23,24]. A major advantage of using L-PRF is the ease of how it can be obtained from the patient’s own blood, making it very suitable for clinical applications [25]. It is an autologous biomaterial that has several advantages in terms of biocompatibility and safety. A substantial amount of research has been performed on the growth factor release profile of different platelet concentrates including L-PRF, but aside from this, not many of its biological features are fully understood [21,22]. The secretome of L-PRF originates from activated leukocytes and especially platelets are cells that store high amounts of key growth factors in their cytoplasmic granules, such as platelet-derived growth factor (PDGF), transforming growth factor-β1 (TGF-β1), vascular endothelial growth factor (VEGF), insulin-like growth factor-1 (IGF-1), epidermal growth factor (EGF), and also several neurotrophic factors [26,27,28]. These mediators, and also the supportive fibrin matrix, are able to stimulate cell proliferation, matrix remodeling, and angiogenesis, thereby affecting the healing and regeneration of injured tissues mainly in the acute phase after administration [29,30]. L-PRF has been used extensively in the craniofacial and endodontic field, and most clinical studies indicate that it improves bone healing and periodontal tissue regeneration [31,32,33,34,35,36,37]. In addition, L-PRF membranes have been used for cartilage and joint repair [38] and also to treat complex skin wounds and ulcers that are often seen in diabetic patients or after trauma (e.g., skin burns) [39,40,41,42]. Increasing evidence in the literature suggests that platelet concentrates can also have positive effects on peripheral nerve repair (reviewed in [43]), possibly by redirecting Schwann cells (the main glia in the PNS) towards nerve repair cells [44,45,46]. However, the mode of action and direct effect of platelet concentrates and L-PRF on peripheral neurons needs to be further elucidated in order to better understand the true potential of L-PRF-derived therapeutics in neuroregeneration. Moreover, the effect of platelet concentrates on neuronal cell functions in the CNS is unexplored. Previously it has been shown that fibrin-based biomaterials containing growth factors enhance neuronal fiber sprouting in vitro or after spinal cord injury [47,48,49], but the effect of L-PRF on the CNS is not known.

The current study aimed to investigate the paracrine-mediated effects of the L-PRF secretome on neuronal components of the CNS and PNS in vitro. We give new insights into the L-PRF morphology and show that it slowly releases a plethora of growth factors and signaling molecules that can affect neural cell functions. Functional experiments provided strong evidence that factors released by L-PRF have a beneficial effect on the survival and neurite outgrowth from dorsal root ganglion (DRG) neurons from the PNS. In contrast, the L-PRF secretome had a detrimental effect on primary cortical neurons (pCNs) and suppressed the metabolic activity of neural stem cells (NSCs). To conclude, L-PRF is a potent biomaterial in regenerative medicine that can support nerve regeneration in peripheral tissues.

## 2. Results

### 2.1. L-PRF Consists of a Porous Fibrin Network with Leukocytes and Aggregates of Activated Platelets

**Figure 1:**
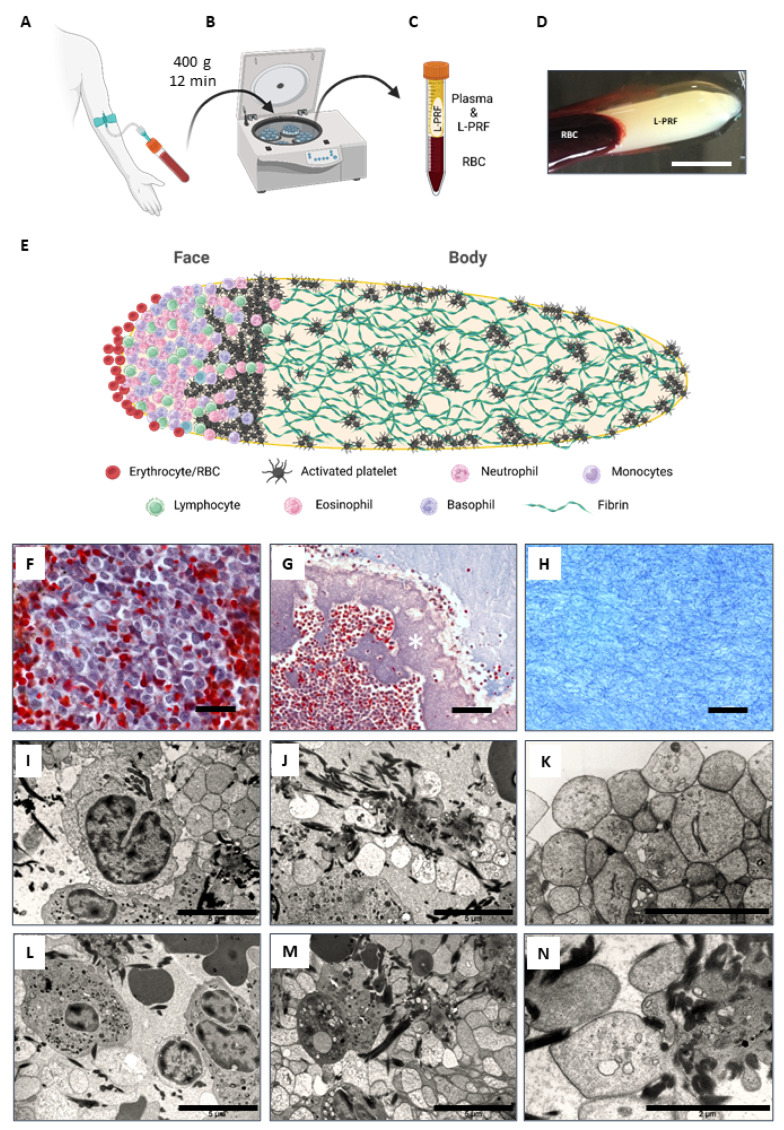
Preparation of L-PRF and its morphological characterization. (**A**–**D**) Illustration of L-PRF preparation. A venous blood sample is taken from healthy individuals and collected in silica-coated tubes (**A**) upon which they are centrifuged (**B**) at 400× *g* for 12 min using an IntraSpin™ centrifuge. The blood coagulates during this centrifugation step generating L-PRF (**C**,**D**). (**E**) Graphical representation of the structure of an L-PRF clot based on our histological findings. Created with BioRender.com. Two different areas can be distinguished, the ‘face’ area containing leukocytes (i.e., neutrophils, monocytes, lymphocytes, eosinophils and basophils), activated platelets, and remaining red blood cells (RBC/erythrocytes), and the ‘body’ which mainly contains a dense fibrin network with aggregations of activated platelets spread throughout the clot and on the surface. (**F**–**H**) A Masson’s Trichrome staining was performed on a sagittal section of an L-PRF clot, illustrating a high concentration of leukocytes and RBC in the face area (**F**), a transition zone (white asterisk) of densely packed platelets (**G**), and the main body containing a dense fibrin network with activated platelets (**H**). (**I**–**N**) Ultrastructural TEM analysis confirmed the presence of neutrophils, lymphocytes, eosinophils, and monocytes in the face area and the aggregates of activated platelets and fibrin fibers in the main body. Scale bar: in (**D**) = 1 cm; in (**F**,**H**) = 20 µm; in (**G**) = 50 µm; in (**I**,**J**,**L**,**M**) = 5 µm; in (**K**,**N**) = 2 µm.

**Figure 2:**
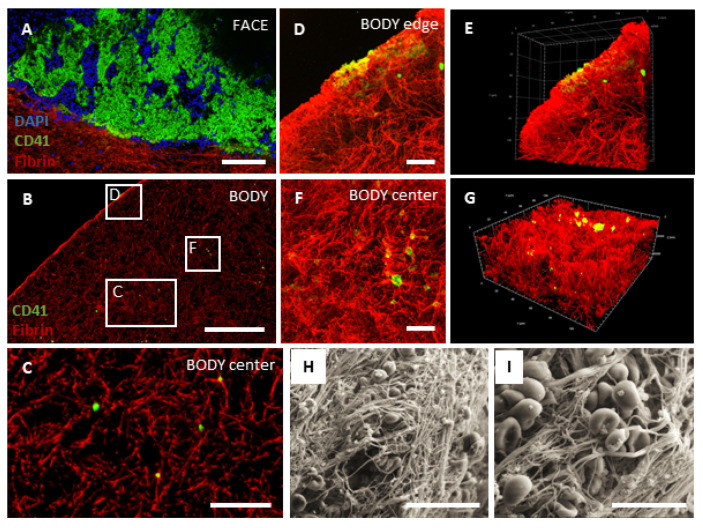
The body of L-PRF consists of a dense and porous fibrin matrix with platelet aggregations. (**A**–**G**) L-PRF was prepared from healthy donors, and immunostaining and confocal imaging for CD41 (platelets, in green) and fibrin (in red) was performed on the face and body region of the clots. A DAPI counterstain (in blue) visualizes the nuclei of leukocytes in the face area. (**A**,**B**) Tile scan images taken at the level of the face (**A**) area and the main body (**B**) of an L-PRF clot. (**C**) Optical slice (0.34 µm thickness) from the area in (**B**) illustrating the fibrin fibers (red) and platelets (green). (**D**,**F**) Maximum intensity projection and (**E**,**G**) 3D representation of a CD41 (in green) and fibrin (in red) stained section at the edge (**D**,**E**) and center (**F**,**G**) of an L-PRF clot. L-PRF is a compact structure and has a porous fibrin matrix that contains platelets, and the density of the fibrin fibers was higher around platelet aggregates. (**H**,**I**) Ultrastructural SEM analysis of an L-PRF clot illustrates the fibrin fibers, red blood cells, platelets, and leukocytes on the surface of an L-PRF clot. Scale bar: in (**A**,**B**) = 200 µm; in (**C**) = 50 µm; in (**D**,**F**) = 20 µm; in (**H**) = 20 µm; in (**I**) = 10 µm.

As a first step, we performed a histological examination to get a better understanding of the morphological structure of L-PRF clots. L-PRF was freshly prepared from healthy donors as illustrated in Figure 1A–D, and sagittal paraffin sections were prepared and stained with a Masson’s Trichrome staining (Figure 1F–H). Two main regions could be distinguished in L-PRF tissue as a result of the clotting and centrifugation process, i.e., a small area with densely packed leukocytes called the ‘face’ area (Figure 1E,F and Figure 2A) and the main ‘body’ that consists of a dense fibrin network with dispersed islands or aggregates of activated platelets (Figure 1E,H and Figure 2B–G). In between these two domains, a transition zone of densely packed and activated platelets is observed, that also reach and branch between the leukocytes in the face area (Figure 1G white asterisks, and Figure 2A). TEM analysis confirmed the presence of leukocytes in the face region at the ultrastructural level, containing predominantly neutrophils, eosinophils, monocytes, and lymphocytes (Figure 1I,L,M). Platelets in L-PRF have an empty appearance because they excrete their granule content upon activation, and only the open canicular system, dense tubular system and glycogen are visible in the cytoplasm (Figure 1K,M,N). Platelet aggregates could be observed throughout the L-PRF clot (Figure 1J,M), and we found that the surface of L-PRF clots was densely covered by activated platelets (Figure 1K). Additionally, the fibrin fiber density was higher in the vicinity of platelet aggregates (Figure 1J,N and Figure 2D–G). Immunostainings for CD41 (platelet marker) and fibrin on L-PRF tissue combined with 3D confocal imaging confirmed the architecture of L-PRF (Figure 2A–G). Ultrastructural SEM images are shown that illustrate the fibrin fibers, red blood cells, platelets, and leukocytes on the surface of an L-PRF clot (Figure 2H,I).

### 2.2. L-PRF Is a Reservoir for a Plethora of Growth Factors That Are Slowly Released over Time

**Figure 3:**
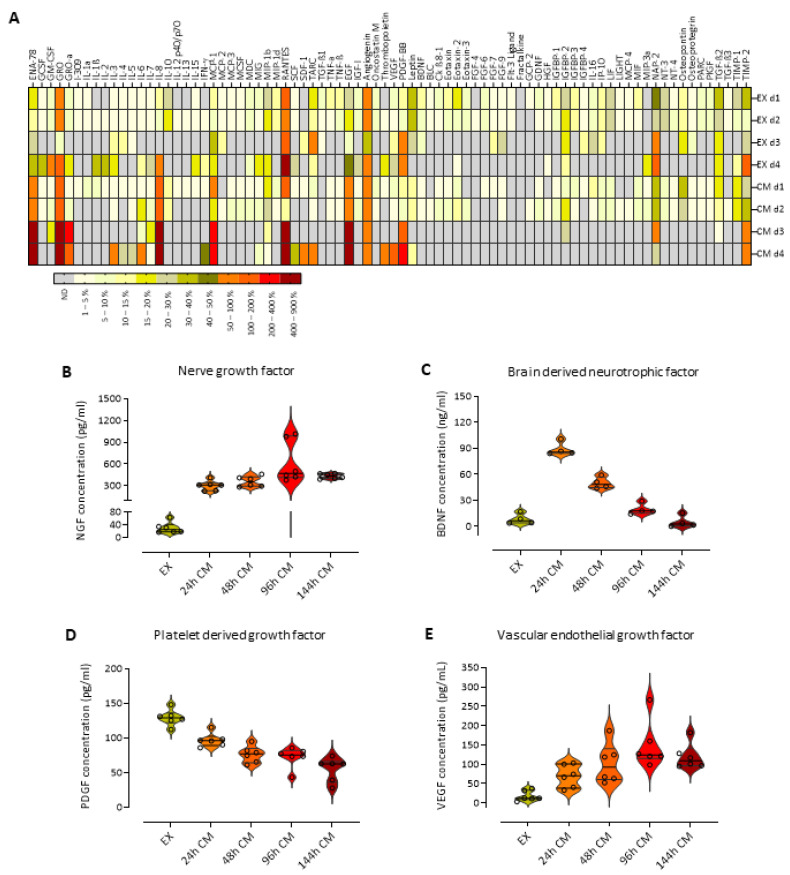
L-PRF contains a plethora of growth factors that are slowly released over time. (**A**) An antibody area was performed to screen the proteins that are present in L-PRF EX and CM (collected after 96 h in culture) from four different healthy donors. A heatmap illustrates the high amount of growth factors that are present in the secretome of the different donors, and some variability is observed between the donors. (**B**–**E**) In order to evaluate NGF (**B**), BDNF (**C**), PDGF (**D**), and VEGF (**E**) release over time, L-PRF clots were incubated in a medium which was collected and replaced with a new medium at 24, 48, 96 and 144 h. Protein levels were quantified using ELISA on the EX and CM collected at the indicated time points (*n* = 4–6 healthy L-PRF donors). L-PRF slowly releases NGF, BDNF, PDGF, and VEGF in time, and even after 6 days in culture high levels of these growth factors were released and detected. Data are shown as violin plots with the mean, minimum, and maximum values.

As a next step, we investigated the secretome of L-PRF and first performed an antibody array to obtain a more general screening of the mediators that are released from the clots. The array was performed on L-PRF EX and CM (collected after 96 h total) of four different healthy donors. A heatmap is shown illustrating the factors that are released by L-PRF (Figure 3A), and raw data of this analysis were published by us (see supplementary materials in Ratajczak, Vangansewinkel et al. [50]). Here, we performed a re-evaluation of the data and focused on neurotrophins and other mediators that play a pivotal role in neuronal cell functions. Analysis indicated high protein levels of NT-3, NT-4, GDNF, BDNF, VEGF, PDGF, TGF-β, and also several immunomodulatory cytokines such as IL-6, IL-10, and IL-13. Additionally, other factors that are involved in wound healing processes such as tissue inhibitors of metalloproteinases (TIMPs), monocyte chemoattractant protein-1 (MCP-1), IGF-1, EGF, and stromal cell-derived factor 1 (SDF-1) were strongly detected. It is, however, important to indicate that there was significant variability between the different donors and between the CM and EX. As a next step, we validated and quantified the release of NGF, BDNF, PDGF, and VEGF at different time points from L-PRF clots by means of ELISA (Figure 3B–E). NGF, BDNF, and VEGF protein levels were significantly lower in L-PRF EX compared to their values in the CM (Figure 3B, C, and E, respectively), but the opposite was observed for PDGF with the highest levels being detected in L-PRF EX (Figure 3D). NGF and VEGF protein levels increased gradually in the medium with longer incubation times (Figure 3B,E), while BDNF and PDGF had their highest concentration in the medium early after incubation and gradually decreased in time (Figure 3C,D). The average peak levels in the CM for NGF (623.7 pg/mL) and VEGF (149.2 pg/mL) were observed at 96 h, and for BDNF (89.12 ng/mL) and PDGF (97.04 pg/mL) at 24 h. The corresponding protein levels for NGF, VEGF, BDNF, and PDGF in the L-PRF EX were 30.14 pg/mL, 18.46 pg/mL, 8.453 ng/mL, and 129.4 pg/mL, respectively. These data indicate that L-PRF contains a plethora of growth factors, that are slowly released in time, and even after 6 days in culture high levels of these growth factors were detected.

### 2.3. The Proliferative and Metabolic Activity of NSCs Was Not Affected by L-PRF 

**Figure 4:**
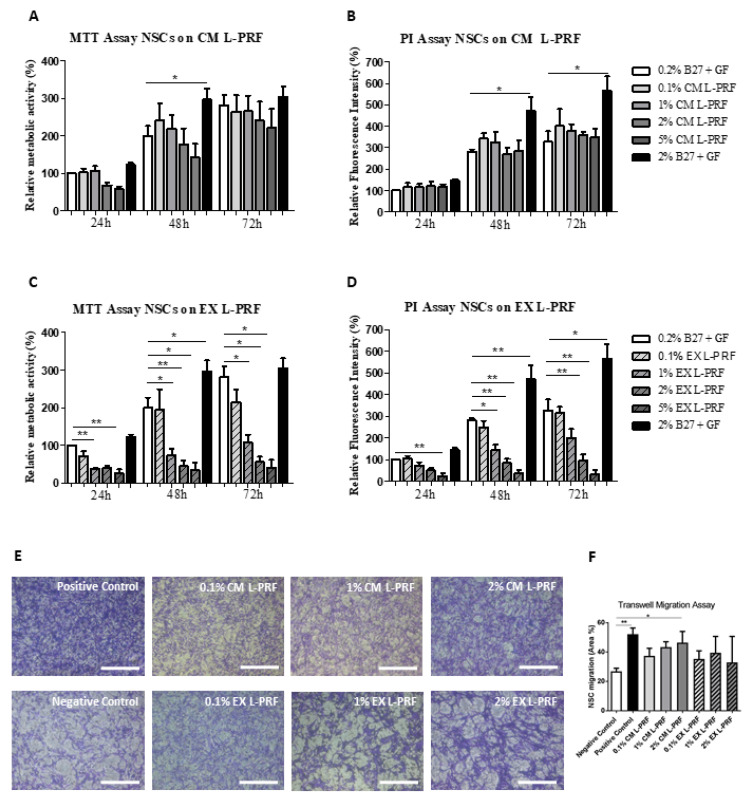
The effect of L-PRF growth factors on the metabolism, proliferation, and migration of NSCs. Neural stem cells (NSCs) were obtained from fetal mouse brains as described in the methods. (**A**–**D**) NSCs were stimulated with different concentrations of L-PRF EX and CM (0.1%, 1%, 2%, and 5%) for 24, 48, or 72 h in vitro, and the metabolic activity and proliferation were measured using an MTT and PI assay respectively. For the control conditions, NSCs were exposed to standard NSC medium supplemented with 20 ng/mL of EGF and bFGF, and with either 0.2% B27 (negative control) or 2% B27 (positive control). L-PRF CM did not have a significant effect on the NSC metabolism (**A**) or proliferative capacity (**B**) over the 72 h testing period. L-PRF EX, on the other hand, significantly decreased NSC metabolism (**C**) and proliferation (**D**) with a dose-response effect. All data were normalized to the values of NSCs exposed to standard NSC medium with 0.2% B27 after 24 h exposure. (**E**,**F**) The chemoattractant properties of L-PRF-derived CM and EX (0.1%, 1%, and 2%) on NSCs were investigated using a transwell migration assay. NSC seeding medium and medium with 2% B27 and 100 ng/mL SDF-1 were used as a negative and positive control, respectively. Representative images are shown of migrated NSCs in transwell inserts stained with 0.1% crystal violet after exposure to the L-PRF secretome (**E**). 2% L-PRF CM significantly stimulated NSC migration, while L-PRF EX had no effect (**F**). Data are expressed as mean + S.E.M. */** *p*-value ≤ 0.05 and 0.01, respectively. Independent experiments were performed with *n* = 3 different NSC lines. *n* = 4–5 healthy L-PRF donors.

NSCs are an invaluable source of neuronal and glial progeny and are known to play an important role in the regenerative response after CNS injury [51,52,53]. As a next step, we explored the effect of L-PRF CM and EX on NSC metabolism and proliferation by means of an MTT and PI assay, respectively. L-PRF CM did not have a significant effect on NSC metabolism or proliferation, although a trend appears to be present that tends to decrease NSC metabolism with higher concentrations of CM (Figure 4A,B). L-PRF EX, on the other hand, had detrimental effects on NSC metabolism and proliferation with increasing concentrations (Figure 4C,D). Only L-PRF EX concentrations as low as 0.1% did not have a significant negative effect over the different time points that were tested.

### 2.4. Growth Factors Slowly Released by L-PRF Act as a Chemoattractant on NSCs

NSC migration towards damaged tissue is one of the key steps in the neuroregenerative response in the injured CNS. Therefore, also the chemoattractive properties of L-PRF EX and CM on NSCs were evaluated. Representative images of migrated NSCs in transwell inserts stained with 0.1% crystal violet after exposure to 0.1%, 1%, or 2% L-PRF EX or CM are shown in Figure 4E. All experimental conditions were compared to the negative control (i.e., standard medium with 0.2% B27 and growth factors), and are expressed as the area percentage covered by migrated NSCs. L-PRF CM was able to significantly stimulate NSC migration but only when 2% CM (*p*-value ≤ 0.05) was used, without a significant difference between 2% CM and the positive control condition (i.e., standard medium with 2% B27 and 100 ng/mL SDF-1) (Figure 4F). Higher concentrations of CM were not evaluated due to the negative trend that was observed in the NSC metabolism when these cells were exposed to concentrations of L-PRF CM ≥ 5%. Regarding L-PRF EX, we did not observe a significant effect on NSC migration (Figure 4F).

### 2.5. L-PRF Growth Factors Had a Detrimental Effect on pCNs and Suppressed Neurite Outgrowth

**Figure 5:**
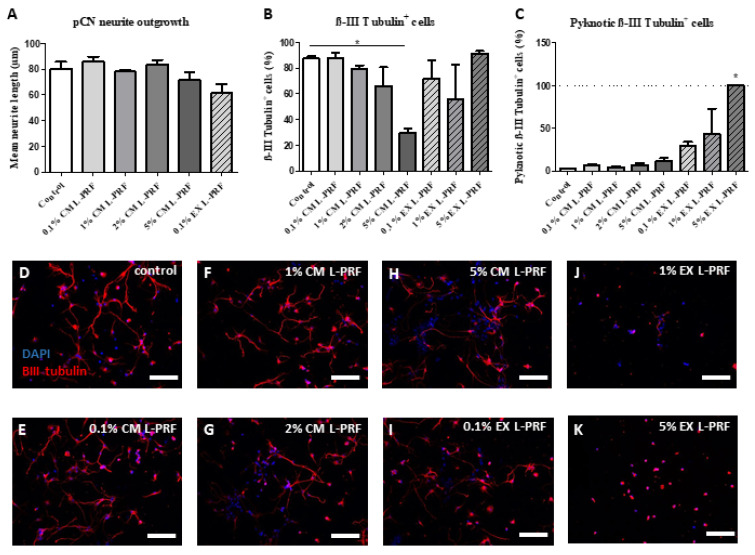
Detrimental effects of L-PRF on the survival and purity of primary cortical neurons. Primary cortical neurons (pCNs) were obtained from fetal mouse brains as described in the methods. These pCNs were stimulated with different concentrations of L-PRF EX and CM (0.1%, 1%, 2%, and 5%) in vitro. Standard pCN medium was included as a control. Neurons and their neurites were visualized via immunocytochemistry for βIII tubulin (green). A DAPI counterstain (blue) was performed to visualize the nuclei. (**A**) L-PRF EX and CM did not enhance neurite outgrowth of pCNs compared to the control condition. (**B**) Exposure to 5% L-PRF CM significantly decreased the number of ßIII tubulin-positive cells in the neuronal culture. (**C**) L-PRF EX did not significantly alter the fraction of ßIII tubulin-positive cells in the culture but had a dose response on the fraction of pyknotic ßIII tubulin-positive cells. (**D**–**K**) Representative photomicrographs of ßIII tubulin stained pCNs after exposure to control medium (**D**), 0.1% CM (**E**), 1% CM (**F**), 2% CM (**G**), 5% CM (**H**), 0.1% EX (**I**), 1% EX (**J**) and 5% EX (**K**). Scale bars in (**D**–**F**) = 50 µm. Data are expressed as mean + S.E.M. * *p*-value ≤ 0.05. Three independent experiments with different batches of pCNs. *n* = 3 healthy L-PRF donors.

To evaluate the effect of the L-PRF components on neurons of the CNS, both neurite outgrowth and neuronal survival of pCNs (87.49% ± 3.052% purity) exposed to L-PRF EX and CM were evaluated (Figure 5). None of the tested concentrations of L-PRF CM and EX was able to stimulate neurite outgrowth of pCNs compared to controls (Figure 5A). Moreover, with increasing concentrations of L-PRF CM, a significant decrease in the fraction of βIII tubulin-positive cells was observed when pCNs were exposed to 5% CM L-PRF for 72 h (Figure 5B). No significant effect in the fraction of βIII tubulin positive cells was observed in pCNs exposed to 0.1%, 1%, or 5% L-PRF EX, but higher concentrations of EX increased the fraction of pyknotic βIII tubulin positive cells in culture, indicating apoptotic neurons (Figure 5C). Due to the pyknotic appearance and deviating morphology of pCNs exposed to 1% and 5% L-PRF EX, quantification of the mean neurite length was not performed for these conditions and 2% L-PRF EX was left out of consideration. Representative images of pCNs on standard medium, 0.1%, 1%, 2%, and 5% L-PRF CM and 0.1%, 1%, and 5% L-PRF EX are shown in Figure 5D–K, respectively.

### 2.6. L-PRF Has a Beneficial Effect on the Survival and Neurite Outgrowth of Peripheral Neurons

**Figure 6:**
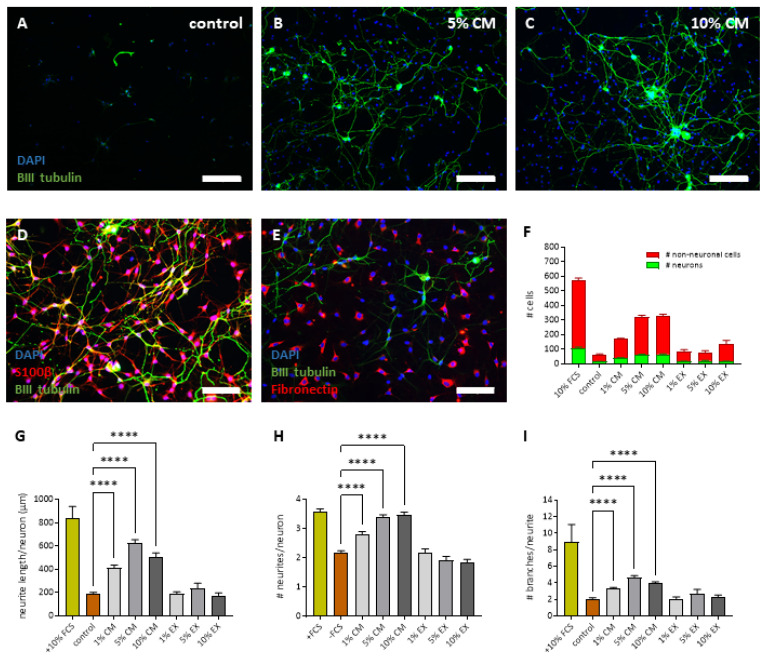
L-PRF growth factors boost the survival and neurite outgrowth from DRG neurons. Rat dorsal root ganglion (DRG) neurons were isolated and cultured for 72 h in the presence of different concentrations of L-PRF EX and CM (1%, 5%, and 10%) in a medium without serum supplementation (trophic deprivation). Medium without serum and medium containing 10% FBS were used as a negative and positive control, respectively. (**A**–**C**) Neurons and their neurites were visualized via immunocytochemistry for βIII tubulin (green). A DAPI counterstain (blue) was performed to visualize the nuclei. Representative photomicrographs are shown for the negative control (medium without serum) (**A**), 5% CM (**B**), and 10% CM (**C**) conditions. (**D**,**E**) Double immunostainings for S100β or fibronectin (in red) with βIII tubulin (in green) confirmed the presence of Schwann cells and fibroblasts in culture. (**F**) The number of non-neuronal cells and neurons in the culture increased with increasing concentrations of L-PRF CM. (**G**–**I**) In addition, the neurite length/neuron (**G**), the number of neurites/neuron (**H**), and the number of branches per neurite (**I**) significantly increased with increasing concentrations of L-PRF CM. Scale bar: in (**A**–**C**) = 200 µm; in (**D**–**E**) = 100 µm. Data are presented as the mean + S.E.M. **** *p*-value ≤ 0.0001. Three independent experiments. *n* = 5–6 healthy L-PRF donors.

As a last step, we explored the neuroprotective and neurite growth-promoting effects of the L-PRF secretome on PNS neurons that were cultured under serum-deprived conditions. For this purpose, rat DRG neurons were isolated and cultured for 72 h in the presence of different concentrations of L-PRF EX and CM (1%, 5%, and 10%, *n* = 6 healthy L-PRF donors) in a medium without serum supplementation. This serum-deprived medium was used to create stress conditions that cause neuronal cell death in culture (trophic deprivation), and was also included as a control condition. Medium containing 10% FBS on the other hand was used as a positive control. Immunofluorescence for βIII tubulin was used to visualize the neurons and their neurites. Representative images of DRG neurons that were grown in serum-free medium (negative control), or medium supplemented with 5% or 10% L-PRF CM are displayed in Figure 6A–C. We found that the number of non-neuronal cells and neurons in the culture increased with increasing concentrations of L-PRF CM (Figure 6F). L-PRF EX did not affect the number of cells in the culture, so in general no toxic effects of L-PRF were observed. In addition, the neurite length/neuron (Figure 6G), the number of neurites/neurons (Figure 6H), and the number of branches per neurite (Figure 6I) significantly increased with increasing concentrations of L-PRF CM. Growth factors in L-PRF EX failed to have any effect on the neurite outgrowth from rat DRG neurons (Figure 6G–I). When performing immuno double stainings for S100B and fibronectin, we found that also Schwann cells and fibroblasts were present in the DRG cultures (Figure 6D,E), and therefore indirect effects of L-PRF on these cell types cannot be excluded. To conclude, L-PRF contains protective proteins that improve the survival and neurite outgrowth of PNS neurons under trophic deprivation.

## 3. Discussion

L-PRF is gaining interest in the field of regenerative medicine. The major advantage of this product is that it can be prepared from the patient’s own blood (i.e., personalized medicine) in a cost-effective way without any additional biochemical modifications. This autologous biomaterial is already being used in the clinic for several applications, mainly intra-oral handlings such as for periodontal tissue regeneration and bone repair [31,32,33,34,35], to treat cranial defects [54], for endonasal skull base surgery [55], but also for wound healing of the skin, all with encouraging results. The underlying mechanisms of L-PRF-mediated tissue repair are generally attributed to its ability to deliver high concentrations of autologous reparative factors. When looking at the known mediators individually (such as VEGF), for several of them it was proven that they play a role in CNS [56,57,58,59] and PNS [60,61,62,63,64] repair, but the effects of L-PRF in the field of neuroregeneration are far from clear. To justify the use of L-PRF in the clinic for enhanced tissue repair in the CNS and PNS, additional preclinical research is mandatory, as its effect on these tissues is largely unknown and an autologous biomaterial as L-PRF might even be bio-incompatible for these applications [65]. Therefore, the main goal of this study was to explore the paracrine effects of the L-PRF secretome on neuronal components in vitro, with a focus on NSCs and pCNs as part of the CNS and DRG neurons from the PNS. 

As a first step, we aimed to examine the morphology of L-PRF by performing electron microscopy and 3D confocal imaging, which is important to better understand its biomaterial applicability. Our histological data confirms the findings from the literature and show that L-PRF clots contain leukocytes and activated platelets that are trapped in a high-density fibrin network. Importantly, the matrix is porous and consists mainly of fibrin but other adhesion molecules (e.g., fibronectin) are also present in natural fibrin clots [66] that can support the attachment and infiltration of cells from its surroundings after transplantation in vivo, such as endothelial cells and blood vessels, fibroblasts, immune cells, and also tissue-specific cells. Moreover, we observed that the fibrin fiber density was higher around islands or aggregates of activated platelets because thrombocytes release fibrinogen including other clotting factors that immediately become activated in their surroundings and platelets also strongly bind to fibrin for example via integrin αIIbβ3 [67,68]. In contrast to PRP type of products, L-PRF only exists in a solid-state form and cannot be injected or used like fibrin glues [69]. However, it is important to note that an injectable type of platelet-rich fibrin (i-PRF) has been developed in the past years [70,71]. Due to the strong fibrin matrix, L-PRF can only be handled as a solid material, and this will predetermine the therapeutic applications for which it can be used. Another important advantage of the fibrin matrix is its capacity to bind and contain growth factors for a certain amount of time after administration in vivo [24,30], and therefore it can have superior properties over other types of PRP in terms of tissue repair. 

As a next step, we determined the secretome and we were specifically interested in the neurotrophin content in the different fractions of L-PRF, namely the exudate (EX) which contains factors that are immediately released from the clots and also the conditioned medium (CM) that contains mediators that are slowly released. An antibody array was performed, and our data demonstrate that an abundance of mediators is present within both the L-PRF EX and CM fraction, which confirms the findings from the literature [24,30,50]. Then, ELISA analysis on EX and CM collected at different time points demonstrated that BDNF, NGF, PDGF, and VEGF are slowly released in time and even after 6 days in culture high protein levels of these growth factors were detected. It is expected that many other factors present within L-PRF clots are released in the same way, and this implies that L-PRF can exert effects in different phases of tissue repair and not only in the acute phase after administration. It is important to point out that from the screening it became clear that there was some considerable donor to donor variability. This may suggest that for a certain treatment or application in the clinic, different responses can be expected between different patients and that maybe not all patients would benefit in the same way from L-PRF treatment. It is known already that the patient’s medical history, such as diabetes and the use of anticoagulant treatment, can influence the mechanical and healing properties of L-PRF [72]. 

As a final step, we performed functional experiments with L-PRF mediators from both the EX and CM fraction to determine their effect on neuronal cell functions and to get a better understanding of their neuroregenerative potential. We observed that the L-PRF secretome had a detrimental effect on the normal physiology of pCN and it suppressed the metabolic activity of NSCs. From a clinical perspective, however, it is important to note that minor positive effects of L-PRF on NSC migration and pCN protection were observed but only with low concentrations of L-PRF CM. Therefore, it can be postulated that the inflammatory mediators within the L-PRF secretome that have detrimental effects on neuronal cell survival, such as TNF-α and IL-1β, exert their neurotoxic effect within higher concentration ranges of L-PRF CM. This could mask the potential beneficial effect of repair-promoting mediators, although the exact composition of L-PRF EX and CM remains to be determined via mass spectrometry for example, and is crucial in understanding L-PRF-mediated effects after in vivo transplantation [73]. L-PRF has been used for treating skull base defects in the vicinity of the blood–brain barrier (BBB) [55]. Moreover, Theys et al. used L-PRF membranes to facilitate dural closure in cranial and spinal neurosurgery [74]. Although no adverse effects were observed in these studies, our data suggest that caution must be taken in using L-PRF this close to the CNS. Cerebrospinal fluid leakage is often seen in these conditions and is an indication of BBB disruption, which can lead to L-PRF secretome leakage into the CNS. 

In contrast to the CNS, the PNS is not limited by a BBB but by a less strict blood nerve barrier, and therefore, it can be postulated that L-PRF or blood-derived factors have different effects on neuronal cells from the PNS. There are several other discrepancies between the CNS and PNS, and the cells within each compartment (e.g., cerebral vs. peripheral neurons, different glial cells) may respond differently in response to certain (blood-derived) mediators. For example, in contrast to CNS glutamate excitotoxicity, glutamate stimulates intracellular signaling and migration of Schwann cells in a rat nerve-crush injury model [75], although DRGs remain susceptible to glutamate-induced excitotoxicity [76]. Encouraging results for PNS nerve regeneration come from studies using platelet-rich fibrin (without leukocytes) and PRP in a rat sciatic nerve injury model, although no significant improvement was observed in the histomorphometric analysis [44]. For PRP it was shown that it improves remyelination and functional recovery after traumatic nerve injury [44,45]. Moreover, it was observed that PRP directly stimulates Schwann cell proliferation, and migration and it increases the release of neurotrophins by these glial cells, thereby affecting healing in the injured PNS [46]. However, the precise mechanism of action and the direct effect of L-PRF on peripheral neurons was unknown until now. Here, we explored the effect of L-PRF EX and CM on DRG neurons from the PNS. We provided strong evidence that factors released by L-PRF have a direct beneficial effect on the survival and neurite outgrowth from DRG neurons. This implies that L-PRF can be used as a biomaterial to improve peripheral nerve repair and that it can support also nerve fiber growth and innervation in other types of tissue repair, such as skin wound healing or tooth implant surgeries. L-PRF was used before in a split-mouth study for the healing of post-extraction sockets, and although post-operative pain was attenuated no data are available on nerve regeneration in this clinical study [34]. Roth et al. provided the first suggestive evidence that L-PRF treatment had a similar effect on the functional outcome after traumatic nerve injury as an autograft, which is the gold standard therapy in the clinic [77]. More recent publications by Vares et al. [78] and Neves Atti et al. [79] also suggest positive effects of L-PRF treatment in a rat model for peripheral nerve injury. However, care should be taken and additional preclinical research is necessary to understand the applicability of L-PRF for improving nerve tissue regeneration in the clinic. In interpreting the findings of this study, it is important to point out that human L-PRF was used to stimulate rodent cells. Although there is a lot of homology in structure and function between the human and rodent form of certain growth factors, such as BDNF and NGF, there are discrepancies in their mode of action as well [80,81]. Therefore it is important to also validate the effect of L-PRF in the future on complete human neural cell culture systems before going to the clinic.

## 4. Materials and Methods

### 4.1. Generation and Processing of Leukocyte- and Platelet-Rich Fibrin (L-PRF)

L-PRF was obtained from healthy volunteers (aged 25–37, *n* = 7, male and female) with written informed consent, and isolation was approved by the Medical Ethical Committee of the University Hospital of Leuven and Hasselt University (S58789/B322201628215/I/U). L-PRF was prepared according to the guidelines of the IntraSpin™ centrifuge (Intra-Lock International, Boca Raton, FL, USA). Briefly, 9 mL venous blood samples were collected in glass-coated tubes (VACUETTE^®^ Z Serum Clot Activator Tubes, Greiner Bio-One, Kremsmünster, Austria) and spun down immediately at 400 g (2700 rpm) for 12 min. The blood coagulates during this centrifugation step, and afterward the resulting L-PRF clots were collected and red blood cell remnants were removed (Figure 1A–D).

### 4.2. Preparation of Exudate and Conditioned Medium from L-PRF 

For the preparation of conditioned medium (CM), L-PRF clots were incubated in 6 mL of serum-free Minimal Essential Medium (MEM, Invitrogen, Waltham, MA, USA) for neural stem cell (NSC) experiments, Neurobasal-A medium (Sigma-Aldrich, St-Louis, MO, USA) for primary cortical neuron (pCN) experiments, or Dulbecco’s Modified Eagle’s medium (DMEM, Sigma-Aldrich) for dorsal root ganglion (DRG) neuron experiments, all supplemented with 100 U/mL Penicillin (Sigma-Aldrich) and 100 μg/mL Streptomycin (Sigma-Aldrich) (1% P/S). After 96 h, the medium was collected, centrifuged for 6 min at 300× *g*, sterile filtered (Filtropur S0.2, Sarstedt, Nümbecht, Germany) and stored at −80 °C until further usage. To collect exudate (EX), freshly prepared L-PRF clots were transferred to a sterile box (Xpression™ Fabrication Box, Intra-Lock, Boca Raton, FL, USA) and pressed into thin membranes, thereby releasing the exudate. The resulting exudate was also collected, filtered, and stored at −80 °C until further usage.

### 4.3. (Immuno)Histological Characterization

For the general histological characterization, L-PRF samples were fixed in 4% paraformaldehyde (PFA) in phosphate-buffered saline (PBS) overnight at 4 °C and routinely embedded in paraffin as previously described [82]. Next, 7 µm thick sagittal sections were cut and after deparaffinization and rehydration, they were colored using Masson’s Trichrome staining. The stained images were evaluated using a photomicroscope equipped with an automated camera (Nikon Eclipse 80i, Nikon Co., Tokyo, Japan).

For immunofluorescent staining, whole L-PRF tissue (body and face area separately, see Figure 1D,E) was blocked with 10% general protein block (Dako, Glostrup, Denmark) and permeabilized with 0.05% Triton X-100 in PBS for 30 min at room temperature (RT). Then, the following primary antibodies were incubated overnight at 4 °C in a humidified chamber: rabbit polyclonal anti-fibrinogen (1:200; ab34269, Abcam, Cambridge, UK) and mouse monoclonal anti-CD41 (for platelets, 1:200, ab11024, Abcam). Following repeated washing steps with PBS, L-PRF tissues were incubated with Alexa-labeled secondary antibodies for 1 h at RT, namely goat anti-mouse IgG Alexa 488, goat anti-rabbit IgG Alexa 647 (dilution 1:250; all secondary antibodies were obtained from Invitrogen). The specificity of the secondary antibody was verified by including a control staining in which the primary antibody was omitted. A 4,6-diamino-2-phenylindole (DAPI, Invitrogen) counterstain was performed to reveal cellular nuclei. Images were taken using a Zeiss LSM 880 (Carl Zeiss, Jena, Germany) mounted on the rear port of an Axio Observer Z.1 inverted microscope and equipped with a 40× water immersion objective (LD C-Apochromat 40×/1.1 W Korr M27, Carl Zeiss). 

Whole L-PRF tissues (face and body area, Figure 2A,B) were imaged by means of z-stack and tile scan imaging with 10% overlap to enable the stitching of the recorded tiles. Image size and pixel density were adjusted to acquire resolution-limited images (108 × 108 nm pixels, 5 µm z slices). 3 laser lines were used for excitation: 488 nm (Argon-ion laser), 633 nm (HeNe laser), and a femtosecond pulsed laser (MaiTai DeepSee, Spectra-Physics, Milpitas, CA, USA) tuned to a central wavelength of 810 nm. A 488/543/633 nm dichroic and a 690 nm short pass dichroic were used as main beam splitters. The resulting fluorescence was collected using a Quasar 32 channel GaAsP spectral detector array selecting 2 different spectral bands: 490–588 nm, 646–694 nm, and channel one of the BiG.2 GaAsP detector in non-descanned detection mode with a BP 380–430 emission filter. In addition, super-resolution z stack images were acquired using the Airyscan module of the LSM880. Image size and resolution were optimized according to instructions of the manufacturer (54 × 54 nm pixels, 0.36 µm z slices). 2 laser lines were used for excitation: 488 nm (Argon-ion laser) and a 633 nm (HeNe laser). A 488/543/633 nm dichroic was used as the main beam splitter. The resulting fluorescence was collected using a dedicated Airyscan detector selecting 2 different spectral bands using the filters: BP 555–620 nm + LP 645 nm and BP 420–480 nm + BP 495–550 nm. Airyscan processing was performed in ZEN black using the auto filter strength. The microscope and acquisition configuration were controlled with ZEN Black 2.6 SP1.

### 4.4. Ultrastructural Analysis: Transmission and Scanning Electron Microscopy 

L-PRF samples were fixed with 2% glutaraldehyde (Laborimpex, Brussels, Belgium) in 0.05 M cacodylate buffer (pH 7.3; Aurion, Wageningen, The Netherlands) at 4 °C for ultrastructural analysis. 

For Transmission Electron Microscopy (TEM), post-fixation was carried out with 2% osmiumtetroxide (Aurion) for 1 h at 4 °C. Dehydration of the samples was performed in ascending concentrations of acetone. Afterward, the dehydrated samples were impregnated overnight in a 1:1 mixture of acetone and araldite epoxy resin. Next, the samples were embedded in Araldite epoxy resin at 60 °C and were cut in slices of 70 nm with a Leica EM UC6 microtome. The slices were transferred to 0.7% formvar-coated copper grids (Aurion). Afterward, the samples were contrasted with 0.5% uranyl acetate and lead citrate (Laurylab, Saint-Fons Cedex, France) using a Leica EM AC20. Analysis was performed with a Philips EM208 S electron microscope (Philips, Eindhoven, The Netherlands) equipped with a Morada Soft Imaging System camera with iTEM-FEI 4.07 software (SIS, Olympus, Tokyo, Japan). 

For Scanning Electron Microscopy (SEM), L-PRF clots were transferred through a gradual ethanol gradient (25, 50, 70, 90%, 2 × 100%, for 10 min each), before being critical point dried with carbon dioxide. Subsequently, specimens were mounted on 12 mm aluminum stubs, and sputter coated with a ∼5 nm thick gold/palladium layer (JEOL JFC-1200 Fine Coater, JEOL, Tokyo, Japan). Images were made with a JEOL JSM-5600 LV (JEOL, Tokyo, Japan) under a high vacuum operated at 10–30 kV.

### 4.5. Secretome Analysis: Antibody Array and Enzyme-Linked Immunosorbent Assay

For screening, a Human Cytokine Antibody Array (ab133998, Abcam, Cambridge, UK) was performed on L-PRF CM (collected after 96 h total) and EX of four different healthy donors at a protein concentration of 10 mg/mL according to the manufacturer’s instructions (Thermo Scientific, Erembodegem, Belgium). Protein concentrations of the samples were determined with a bicinchoninic acid assay (BCA, Thermo Fisher Scientific, Waltham, MA, USA) following the user manual. Relative pixel density was measured using ImageJ/Fiji software 1.53 (NIH, Bethesda, MD, USA) to compare relative protein levels between L-PRF EX and CM. This analysis was previously performed by our group [50,83]. This study reinterpreted the data of these arrays focusing on the neurotrophic factors and other growth factors present in both L-PRF subfractions that may affect neuronal cell functions. In addition, an Enzyme-Linked Immunosorbent Assay (ELISA) (Raybiotech, Peachtree Corners, GA, USA) was performed to quantify and verify vascular endothelial growth factor (VEGF), brain-derived neurotrophic factor (BDNF), nerve growth factor (NGF) and platelet-derived growth factor (PDGF) protein levels in L-PRF EX and CM collected at 24, 48, 96, and 144 h to evaluate the release of these growth factors from the clot over time.

### 4.6. Isolation and Culture of Rat Dorsal Root Ganglion Neurons

Dissociated dorsal root ganglion (DRG) cultures were prepared from adult (200–250 g) Sprague-Dawley rats as previously described [84]. In order to obtain DRGs, rats were sacrificed and the spinal column was excised and divided in half in the sagittal plane to expose the spinal cord. Next, the spinal cord tissue was removed to expose the DRGs in the intervertebral foramen. Under a dissecting microscope, the DRGs were removed and placed in a petri dish containing Dulbecco’s modified eagle’s medium (Gibco, Thermofisher Scientific, Merelbeke, Belgium) supplemented with 10% fetal bovine serum (FBS, Biowest^®^, Nuaillé, France) and 1% P/S. Around 25–30 DRGs were collected from the thoracic and lumbar regions, and cleaned by removal of the nerve roots and connective tissue. Next, the DRGs were enzymatically dissociated by incubation in 0.125% collagenase type IV solution (prepared in growth medium without serum) at 37 °C for 90 min. Afterward, the explants were mechanically dissociated (triturated) with a glass Pasteur pipette, and the collagenase was removed by two spin washes in a growth medium at 400 g for 5 min. The pellet was resuspended in DMEM growth medium supplemented with 10% FBS, 1% P/S, and 10 µM cytosine ß-D-arabinofuranoside (Ara-C, Sigma-Aldrich) to deplete the non-neuronal cells (i.e., dividing satellite glial cells and fibroblasts). Cells were plated in poly-L-Iysine coated (50 μg/mL, Thermofisher Scientific) T-75 culture flasks and incubated at 37 °C and 5% CO_2_ in a humidified atmosphere for 24 h before use. 

### 4.7. Isolation and Culture of Mouse Neural Stem Cells and Primary Cortical Neurons 

Mouse neural stem cells (NSCs) were isolated and cultured from fetal mouse brains as previously described [83,85,86]. At gestational days 14–15, pregnant C57BL/6 mice (Janvier, St. Berthevin Cedex, France) were sacrificed by cervical dislocation, and the fetuses were removed from the abdomen. Subsequently, the brains were removed and cut into small fragments in cold (4 °C) PBS with 1% P/S. The brain fragments were then transferred to a new vial and centrifuged for 8 min at 200× *g*. Afterward, the supernatant was removed and incubated with 0.2% collagenase A (Roche, Basel, Switzerland) and DNase-I (2000 Kunitz units/50 mL) in PBS for 1.5 h at 37 °C. The obtained dissociated tissue was subsequently washed and resuspended in Neurobasal-A medium supplemented with 1% N2 (Invitrogen), 10 ng/mL epidermal growth factor (EGF), and basic fibroblast growth factor (bFGF) (both from ImmunoTools, Friesoythe, Germany), and 1% P/S. The cell suspension was then rinsed through a 70 μm cell strainer and transferred to an uncoated culture flask to allow neurosphere formation and removal of unwanted, plastic adherent cells. The neurospheres were allowed to grow for 4–5 days at 37 °C in a humidified atmosphere containing 5% CO_2_, and the growth factors were replenished every other day. Neurosphere collection occurred by centrifugation at 200× *g* for 6 min followed by dissociation with Accutase^®^ for 5 min. Subsequently, the obtained NSCs were seeded and grown in T-25 culture flasks coated with 5 μg/mL fibronectin (R&D Systems, Minneapolis, MN, USA) in Neurobasal-A medium supplemented with 2% B27 without vitamin A (Vit A, Invitrogen), 10 ng/mL EGF and bFGF, 2 mM L-glutamine, and 1% P/S, which will be referred to as standard NSC medium. The culture medium was changed every 3 to 4 days and cells were subcultured when 70–80% confluence was reached. NSCs were harvested by incubation with Accutase (37 °C) and centrifuged for 5 min at 300× *g* before further use. 

Primary cortical neurons (pCNs) were isolated and maintained in culture from fetal mouse brains by an adapted protocol [83,87]. At gestational days 17–18, pregnant C57BL/6 mice (Janvier, France) were sacrificed by cervical dislocation, and the fetuses were removed from the abdomen. Subsequently, the brains were removed and put into preheated Hank’s balanced salt solution (HBSS, Invitrogen) supplemented with 7 mM 4-(2-hydroxyethyl)-1-piperazineethanesulfonic acid (HEPES, Invitrogen) and 1% P/S. Afterward, the meninges were carefully removed with forceps under a Leica S6E stereomicroscope (Leica Microsystems, Wetzlar, Germany) and the cortex was dissected discarding the hippocampus, thalamus, and striatum. The obtained cortices were collected in HBSS/HEPES and incubated with 0.05% trypsin for 15 min at 37 °C. The cortices were then washed three times with MEM medium (Invitrogen) supplemented with 10% horse serum (Invitrogen), 0.6% glucose, and 1% P/S and were mechanically dissociated. The acquired cell suspension was centrifuged for 8 min at 300× *g*, and the cells were resuspended in a supplemented MEM medium and rinsed through a 70 μM cell strainer to obtain a single-cell suspension. pCNs were grown in T-25 culture flasks coated with 20 μg/mL poly-D-lysine and incubated at 37 °C and 5% CO_2_ in a humidified atmosphere before further use.

### 4.8. Metabolic Activity and Proliferation Assay 

The relative metabolic and mitotic activities of NSCs exposed to L-PRF EX and CM were evaluated by means of a 3-(4,5-dimethylthiazol-2-yl)-2,5-diphenyltetrazolium bromide (MTT) and propidium iodide (PI) assay, respectively. The latter takes the amount of PI intercalating between DNA as a measure for the total number of cells. NSCs were seeded in triplicate at a density of 3 × 10^4^ cells/cm^2^ in 96-well plates coated with 5 μg/mL fibronectin (R&D Systems) in standard NSC medium as described above. After 24 h, the medium was discarded and NSCs were exposed to 0.1%, 1%, 2%, and 5% L-PRF CM or EX for 24, 48, or 72 h. To ensure NSC viability, 0.2% B27 without Vitamin A and 10 ng/mL EGF and bFGF were added to all experimental L-PRF conditions. For the control conditions, NSCs were exposed to standard NSC medium supplemented with 20 ng/mL of EGF and bFGF, and with either 0.2% B27 (negative control) or 2% B27 (positive control) without Vitamin A. To perform the MTT assay at the indicated time points, the medium was removed and 500 μg/mL MTT was added. After 4 h of incubation, the MTT-containing solution was removed and 0.01 M glycine in DMSO was added to the wells in order to dissolve the formed formazan crystals. The absorbance was measured at 570 nm and corrected for the background signal at 655 nm with an iMark™ Microplate Reader (Bio-Rad, Hercules, CA, USA). In order to perform the PI assay, the medium was removed at different time points and a 1:1 ratio of lysis buffer (Reagent A100, Chemometec, Allerod, Denmark) and stabilizing solution (Reagent B, Chemometec, Allerod, Denmark) containing 10 µg/mL PI was added and incubated at RT in the dark for 15 min. Afterward, the solution was transferred to a black 96-well plate and fluorescence was excited at 540 nm and measured at 612 nm with a Fluostar Optima (BMG Labtech, Ortenberg, Germany).

### 4.9. Transwell Migration Assay

Tissue culture inserts (ThinCert™, 8 μm pore, Greiner Bio-One, Vilvoorde, Belgium) were seeded with NSCs (5 × 10^4^ cells/insert) in Neurobasal-A medium supplemented with 0.2% B27 without Vit A, 2 mM L-glutamine, 1% P/S, 10 ng/mL bFGF and 10 ng/mL EGF (i.e., standard NSC medium). The bottom compartment of the well plate contained the same seeding medium with different concentrations of L-PRF EX or CM (0.1%, 1%, 2%). Conditions with only the seeding medium or seeding medium with 2% B27 and 100 ng/mL SDF-1 (Immunotools) were used as a negative and positive control, respectively. After keeping the cells in culture for 24 h, the transmigrated cells were fixed with 4% PFA followed by staining with 0.1% crystal violet for 10 min. Migrated cells were at the bottom side of the insert; thus, the upper sides of the inserts were swapped with cotton buds. From each insert, four pictures of the bottom side were taken on 10× magnification with a Nikon eclipse TS100 inverted microscope (Nikon Co., Tokyo, Japan) with a Jenoptik ProgRes C3 camera (Jenoptik, Jena, Germany). The area occupied by migrated cells was quantified using the AxioVision software 4.6 (Carl Zeiss Vision, Aalen, Germany). 

### 4.10. Immunocytochemistry and Neurite Outgrowth Analysis

pCNs and DRG neurons were grown on glass coverslips at a density of 2.5 × 10^4^ cells/cm² and cultured under standard conditions as described above. After 24 h, the medium was replaced with the experimental conditions with varying concentrations of L-PRF EX and L-PRF CM. For the pCNs, their standard medium was supplemented with 0.1, 1, 2, or 5% L-PRF EX or CM, and as a control, a condition with only standard pCN medium was included. For the DRG neurons, the medium was supplemented with L-PRF EX (5%, 10%) or CM (5%, 10%, 50%). Control conditions receiving either 10% FBS (positive) or 0% FBS (negative) were included as well. 

After 72 h of stimulation, the neuronal cultures were fixed with 4% PFA and immunocytochemistry was performed for ßIII tubulin to visualize neurons and their neurites, according to standardized protocols [88]. Briefly, cells were blocked with 10% general protein block (Dako) and permeabilized with 0.05% Triton X-100 in PBS for 30 min at RT. Then, the cells were incubated with a primary mouse monoclonal anti-βIII tubulin antibody (clone 2G10, 1/200, Sigma-Aldrich) overnight at 4 °C in a humidified chamber. Following repeating washing steps with PBS, incubation was performed with a goat anti-mouse Alexa Fluor^®^ 488 or 555 antibodies (1:250, Invitrogen) for 1 h at RT. To check the purity of the DRG cultures, immuno double labeling was performed for βIII tubulin and either S100β (Schwann cell marker) or fibronectin (fibroblast marker). The rabbit anti-S100B (1/200, Dako) and rabbit anti-fibronectin (1/200, Abcam) primary antibodies were used in combination with the secondary antibody goat anti-rabbit Alexa Fluor^®^ 555. Negative controls were included, omitting the primary antibody. A DAPI counterstain was performed, and after mounting, images were taken with a Nikon Eclipse 80i microscope and a Nikon digital sight camera DS-2MBWc. 

Quantitative image analyses were performed on original unmodified photomicrographs. Fiji/ImageJ open source software (NIH) and the Neurite Outgrowth (NEO) Assay 6.1 software (DCILabs, Keerbergen, Belgium) were used for the neurite outgrowth analysis of the pCNs and DRG neurons, respectively. For standardization, at least 50 neurites were counted and only clearly distinguishable neurites were measured. The following parameters were examined: number of neurites/neurons, neurite length/neuron, branches/neurite, and total neurite density. Simultaneously, the fraction of ßIII tubulin-positive cells was evaluated to determine the purity of the obtained cultures. To maximize the image readability of the representative images, the contrast and brightness of the stainings were digitally enhanced equally in the corresponding group.

### 4.11. Statistical Analysis

All statistical analyses were performed using GraphPad Prism 5.01 software (GraphPad Software, Inc., San Diego, CA, USA). Datasets were analyzed for normal distribution using the Shapiro-Wilk test. Normally distributed data were tested with one-way ANOVA followed by the Bonferroni post hoc test for multiple comparisons. Non-parametric data were analyzed with the Kruskal–Wallis test followed by Dunn’s test. Differences were considered statistically significant at *p*-values ≤ 0.05. Data were presented as mean + standard error of the mean (S.E.M) unless stated otherwise. The number of L-PRF EX and CM donors used for the specific experiments is stated in the figure legends.

## 5. Conclusions

To conclude, L-PRF is an autologous biomaterial that has promising potential in improving nerve regeneration and innervation of damaged tissues in the periphery because of its direct positive effects on PNS neurons. It is a natural product that provides transient structural support due to its fibrin matrix, and directs the endogenous response of injured tissue toward repair due to the slow release of functional proteins. It is important to emphasize that care should be taken when using it for CNS applications because of its potential neurotoxic effects on cells derived from the CNS.

## Data Availability

The data underlying this article will be shared upon reasonable request by the corresponding author.
